# Non-Diabetic Hypoglycemia: Evaluation and Management in Adults

**DOI:** 10.3390/jcm14134393

**Published:** 2025-06-20

**Authors:** Eugene Looi, Helen M. Lawler

**Affiliations:** Department of Endocrinology, Diabetes and Metabolism, University of Colorado School of Medicine, Anschutz Medical Campus, Aurora, CO 80045, USA; eugene.looi@cuanschutz.edu

**Keywords:** non-diabetic hypoglycemia, Whipple’s triad, insulin-mediated hypoglycemia, non-insulin-mediated hypoglycemia, non-diabetic medications

## Abstract

Hypoglycemia is defined by the presence of Whipple’s triad, which is (1) low plasma glucose concentration, (2) neurogenic and neuroglycopenic symptoms and/or signs, and (3) their resolution with normalization of plasma glucose concentration. Hypoglycemia in adult patients without diabetes is rare and much less common compared to patients with diabetes. Because of its rarity in the general adult population, recognition and treatment may be delayed. Our review provides a comprehensive summary of non-insulin-mediated and insulin-mediated hypoglycemia in adult patients without diabetes. It explores the pathophysiology of various causes of hypoglycemia and reviews diagnostic approaches such as clinical history, key biochemical findings, and other relevant diagnostic modalities that aid in distinguishing among the different causes, from non-insulin-mediated (e.g., critical illness) to insulin-mediated causes (e.g., post-bariatric hypoglycemia). Our aim is to present the most up-to-date information regarding the diagnosis and management of non-diabetic hypoglycemia to increase awareness and understanding of the condition and promote prompt recognition in patients to expedite diagnosis and treatment.

## 1. Introduction

Hypoglycemia is defined by the presence of Whipple’s triad, which is (1) low plasma glucose concentration (2) neurogenic and/or neuroglycopenic symptoms and (3) symptom resolution with normalization of plasma glucose concentration [1]. In adult patients without diabetes, hypoglycemic symptoms tend to occur at an average blood glucose (BG) level of 55 mg/dL [1]. However, unless there is underlying pathophysiology, hypoglycemia prompting medical attention and evaluation is rare due to intact counterregulatory responses in this population. For instance, in a six-year retrospective study in Finland, the rate of hypoglycemia-related emergency medical service encounters for non-diabetic hypoglycemia was only 1.1% (1082/100,000 persons per year) [2].

Due to its rarity in adult patients without diabetes, hypoglycemia in this population may be underrecognized and remains a diagnostic challenge. As such, this will be the focus of this review. The first step is to obtain a thorough history and physical, including use of medications, alcohol, and supplements [1]. It is also important to inquire about any triggers, symptoms, severity, timing, and any relation to food or activity. Symptoms can usually be categorized as neurogenic and neuroglycopenic (Table 1) [3]. Whipple’s triad should be confirmed. Often, it is difficult to obtain plasma BG when symptoms are occurring, and patients are typically given a glucometer or continuous glucose monitor (CGM) to quickly correlate symptoms with BG.

Nevertheless, glucometers and CGMs are instruments that are not without their limitations. The performance of home glucometers should be calibrated according to standards as established by the Food and Drug Administration (FDA) or the International Organization for Standardization (ISO) [4]. These standards require that 95% of readings from a particular home glucometer fall within 15% of laboratory results. This standard was found to rarely lead to errors in insulin dosing [4]. However, a study involving 17 commercially available home glucometers found that only two met the ISO standard and that accuracy decreased when BG was in the hypoglycemic range [5]. As such, when a patient without diabetes reports hypoglycemic symptoms without hypoglycemia (BG < 55 mg/dL) on a home glucometer, health care providers should not only perform a history and physical but also verify the standard to which the glucometer is calibrated and compare it in real time against a professional glucometer. This is because professional glucometers are held to a higher standard of accuracy [4]. If there is continued doubt in the accuracy of the glucometers, providers may recommend that patients undergo observation in a clinic and replicate the setting where hypoglycemia most frequently occurs. When symptoms occur, a laboratory technician that is on hand can then promptly draw a plasma sample before treatment is initiated.

CGMs are becoming increasingly available to patients without diabetes. These devices measure interstitial fluid glucose that has diffused from the capillaries and into the subcutaneous tissue and may indicate a falsely low BG due to device compression, device calibration errors, and skin/soft tissue infections [4,6,7]. Also, low BG alarms are programmed for patients with diabetes where BGs of 60s–70s are normal in patients without diabetes. As such, when an asymptomatic patient without diabetes reports hypoglycemia on CGM, the health care provider can provide some helpful advice and education. Strategies to avoid inappropriately reacting to erroneous interstitial fluid glucose measurements include comparison with capillary BG when there is discrepancy between symptoms and CGM reading, performing CGM calibration when interstitial glucose is stable, and ensuring good skin hygiene [4]. Patients should be made aware that site placement and sleeping position may potentially cause compression, leading to falsely low BGs.

**Figure 1:**
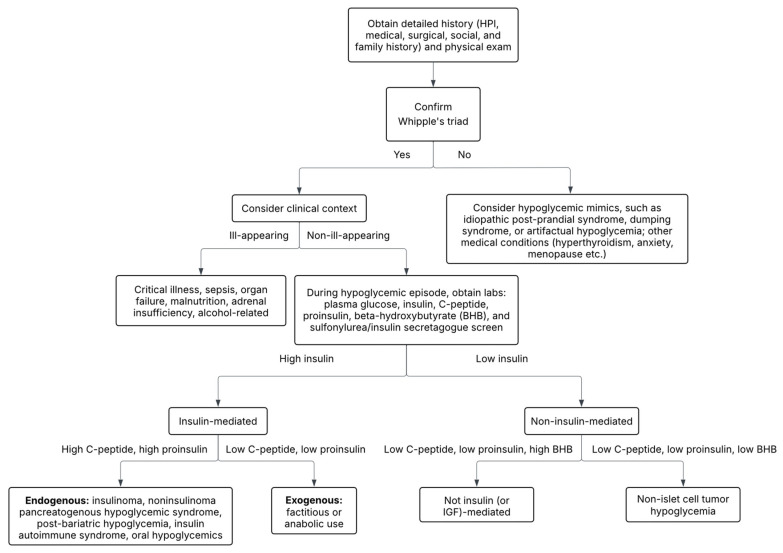
Diagnostic algorithm for non-diabetic hypoglycemia. See Supplemental Figure S1 for detailed step-by-step diagnostic algorithm.

If Whipple’s triad has been confirmed, the differential diagnosis for hypoglycemia is broad and can be classified into two categories to assist with diagnostic accuracy and management: non-insulin-mediated hypoglycemia and insulin-mediated hypoglycemia (Table 2). Another useful approach is to organize hypoglycemia into two groups: patients who appear well (i.e., endogenous hyperinsulinism) and hypoglycemia in patients who appear ill (i.e., critical illness, hormone deficiency, non-islet cell tumor) (Figure 1) [1]. In this review, we will examine non-diabetic hypoglycemia, reviewing various etiologies along with their diagnosis and management.

## 2. Non-Insulin-Mediated Hypoglycemia

### 2.1. Malnutrition and Starvation

Malnutrition is not an uncommon occurrence in economically developed regions. However, the presence of food deserts and insecurity, increasing life expectancy, and rising chronic disease prevalence may be contributing factors to its occurrence. Approximately 5–10% of community-dwelling individuals > 65 years of age and ~50% of nursing home residents experience malnutrition [8]. Patients with chronic medical conditions such as Alzheimer’s disease, obstructive lung disease, cirrhosis, congestive heart failure, or renal failure are at risk for malnutrition—the prevalence of which can range between 20 and 50% in this population [9].

Individuals with poor nutritional status are at increased risk for hypoglycemia [10]. Regardless of comorbid diabetes, malnutrition nearly doubles the risk for hypoglycemia in hospitalized patients [11]. A variety of mechanisms contribute to increased risk, most of which involve players crucial in glycemic homeostasis. Hepatic glycogen and gluconeogenesis are critical to maintain a ready supply of glucose during fasting states, while skeletal muscle glycogen stores serve as an energy source during exertional periods but may also be mobilized during fasting periods [12]. After ~24–48 h of fasting, these reservoirs become depleted [9]. Impaired insulin secretion in the setting of malnutrition, although seemingly protective against hypoglycemia, may also lead to decreased glycogen replenishment, as insulin is a stimulus for glycogenesis [13]. Gluconeogenesis is another physiologic pathway that is disrupted by malnutrition. This results from hepatic glucagon resistance and decreased availability of gluconeogenic substrates, including lactate, glycerol, and alanine [14].

### 2.2. Alcohol-Related Hypoglycemia

Alcohol consumption is another non-insulin mediated cause of hypoglycemia. Overall, hypoglycemia hardly occurs in healthy individuals who consume alcohol. Its occurrence may depend on underlying risk factors such as malnourishment, hepatic dysfunction, and diabetes [15]. Hypoglycemia was found in ~25% of patients with alcohol use disorder presenting with alcoholic ketoacidosis [16]. In addition, another study found that 4% of patients with elevated blood alcohol level of 0.1% were found to be hypoglycemic and the rate of hypoglycemia did not appear to differ between intoxicated vs. non-intoxicated patients [17].

Alcohol contributes to hypoglycemia through a variety of mechanisms. Ethanol is metabolized by alcohol dehydrogenase, which reduces NAD+ to NADH in the oxidative process. The increased NADH level inhibits two primary steps in gluconeogenesis: the conversion of lactate into pyruvate and oxaloacetate into malate [18]. It has been found that 48 g of alcohol, which is a little over three standard drinks, can suppress hepatic gluconeogenesis by as much as 45% [19]. In a healthy individual without risk factors, this usually does not cause issues, as alcohol also stimulates hepatic glycogenolysis [18]. However, in the setting of poor nutritional status or impaired hepatic function, glycogen stores are likely already depleted, leading to an increased risk for hypoglycemia. In addition, in patients with diabetes, studies have found that alcohol ingestion is associated with a reduction in growth hormone levels, which may attenuate counterregulatory responses to hypoglycemia [18]. Furthermore, alcohol intake can impair cognitive function, resulting in decreased awareness of hypoglycemia symptoms, which may exacerbate its danger [20].

Strategies to prevent alcohol-induced hypoglycemia include abstinence, but in cases where abstinence is not chosen, ≤1 drink for females and ≤2 drinks for males are recommended, along with avoidance of alcohol intake after fasting for several hours and binge drinking [21]. Individuals should be counseled to pace their alcohol intake and to consume alcohol with a meal. Such strategies may prevent hypoglycemia and alcohol-related negative outcomes.

### 2.3. Critical Illness-Associated Hypoglycemia

The rate of hypoglycemia in the intensive care setting depends on the definition used, with incidence rates in the first 24 h of admission ranging from 1.5% (if hypoglycemia is defined as a blood glucose < 44 mg/dL) to almost 14% (if hypoglycemia is defined as a blood glucose < 82 mg/dL) [22]. Hypoglycemia in this setting occurs through a variety of mechanisms, which contribute to an imbalance in glucose utilization and production.

In septic shock, underlying dysregulated immune responses produce cytokines in response to infection [23]. This systemic overdrive accelerates glucose utilization that eventually may outpace the body’s gluconeogenic capacity. Cytokines also have been found to inhibit hepatic gluconeogenesis, which further exacerbates this energy mismatch [24]. Limited nutrition is another factor that may prevent replenishment of hepatic glycogen stores. Maintaining adequate nutrition has been associated with improved patient outcomes, and guidelines recommend that nutritional support be initiated within 48 h of admission to the intensive care unit [25].

Although the liver has been well recognized as a major gluconeogenic organ, the kidney is responsible for ~40% of gluconeogenesis in the fasting state. This process occurs in the proximal tubules under hormonal regulation [26]. In the setting of critical illness, acute kidney injury is a common occurrence, with rates up to 67% [27]. Under normal conditions, proximal tubular cells participate in gluconeogenesis and generate ATP via fatty acid oxidation. When there is poor perfusion and decreased oxygen supply, intracellular metabolic pathways shift to glycolysis, an anaerobic but inefficient method of ATP generation. There is some evidence that, while this may be a protective response of the kidney in times of stress, this process may contribute to the development of chronic kidney disease (CKD) [28]. CKD is a risk factor for non-diabetic hypoglycemia, as reflected in significantly higher hypoglycemia rates in patients with CKD compared to patients without CKD (3.46 vs. 2.23/100 patient-months, *p* < 0.0001) [29].

### 2.4. Adrenal Insufficiency

Adrenal insufficiency (AI) is another cause of hypoglycemia. However, the incidence of AI-related hypoglycemia is not well characterized, although the risk appears to be higher in primary compared to secondary AI [30]. In a Japanese study, researchers found that 6% of patients who sought emergency medical care due to hypoglycemia-related symptoms were diagnosed with AI [31]. Although hypoglycemia may not be a common presenting sign of AI, this endocrinopathy should be on the differential when patients present with Whipple’s triad, especially when other AI-associated symptoms are present such as weight loss, fatigue, decreased appetite, nausea, vomiting, abdominal pain, dizziness, and hypotension.

Patients with AI are at increased risk for hypoglycemia due to a variety of reasons. First, glucocorticoids are involved in the counterregulatory hormonal response to hypoglycemia and are required for appropriate adrenal medulla development and function, including the production of catecholamines, which are also involved in the same response [32]. Second, the lack of cortisol may augment insulin sensitivity, leading to increased glucose uptake. As a result, nocturnal fasting hypoglycemia may occur, as insulin sensitivity tends to be highest between the hours of 2–4 a.m. [33]. Lastly, cortisol deficiency has been found to increase glucose oxidation and decrease endogenous glucose production [34]. The mainstay of treatment is to encourage compliance with physiologic glucocorticoid replacement and ensure easy access to simple carbohydrates, parenteral glucagon, and stress-dose steroids for emergency use [32].

### 2.5. Glycogen Storage Diseases

Glycogen storage diseases (GSD) have an incidence of <1 in 20,000). Most are inherited in an autosomal recessive manner and are characterized by impaired glycogenolysis resulting in the glycogen accumulation [12,35,36]. Diagnosis is usually made by DNA analysis or tissue biopsy demonstrating a lack of specific enzymatic activity. This section will only focus on the well-characterized classic GSD types that directly cause hypoglycemia (Table 3).

#### 2.5.1. Glycogen Storage Disease Type 0

Unlike other types of GSDs, GSD type 0 is caused by a lack of—rather than accumulation of—hepatic glycogen. This disorder is caused by either a deficiency in glycogen synthase 1 (enzymatic isoform in cardiac and skeletal muscle; GSD type 0a) or a deficiency in glycogen synthase 2 (GSD type 0b), which catalyzes the formation of an oligosaccharide primer essential for glycogen synthesis in the liver. This disease is characterized by impaired postprandial glycogen storage, leading to postprandial hyperglycemia but fasting ketotic hypoglycemia. Gluconeogenesis is preserved. Management involves avoiding fasting, eating frequent high protein meals to provide gluconeogenic substrates, and consuming uncooked cornstarch to slow glucose absorption.

#### 2.5.2. Glycogen Storage Disease Type Ia and Ib

Also known as von Gierke disease, GSD Type Ia is caused by a deficiency in glucose-6-phosphatase α, which catalyzes the final common step of glycogenolysis and gluconeogenesis—the conversion of glucose-6-phosphate (G6P) into glucose. This impaired glucose production leads to fasting hypoglycemia in infants, hepatomegaly, hypertriglyceridemia, lactic acidosis, and hyperuricemia, which may later lead to the development of chronic nephropathy [12]. GSD Type Ib results from a deficiency in G6P translocase, which is necessary for G6P transport into the endoplasmic reticulum where the final step of gluconeogenesis occurs. This subtype is further characterized by neutropenia, susceptibility to infections, and inflammatory bowel disease. Management involves regular consumption of extended-release uncooked cornstarch (Glycosade) and continuous enteral nutrition. A high protein diet is not recommended. This is due to the biochemical location of the enzymatic defect, which ultimately prevents conversion of amino acid substrates into glucose. Such a diet may predispose to worsening nephropathy.

#### 2.5.3. Glycogen Storage Disease Type III

Also known as Cori or Forbes disease, GSD Type III is caused by a deficiency in hepatic glycogen debranching enzyme (GDE), which is required to hydrolyze the α (1→6) glycosidic bonds present at glycogen branch points. This step is necessary for glycogen phosphorylase to complete glycogen breakdown. GDE deficiency results in accumulation of abnormal glycogen molecules and leads to hepatomegaly, fasting ketotic hypoglycemia, and significantly elevated hepatic transaminases and creatinine kinase. Like GSD type 0, gluconeogenesis is preserved. Management involves prevention of hypoglycemia through frequent high protein meals and consumption of uncooked corn starch.

#### 2.5.4. Glycogen Storage Disease Type VI

Also known as Hers’ disease, GSD Type VI is caused by a deficiency in hepatic glycogen phosphorylase, the principal enzyme in glycogenolysis that produces glucose-1-phosphate, which subsequently is converted to G6P and then into glucose. This accumulation of hepatic glycogen leads to hepatomegaly, fasting ketotic hypoglycemia, but normal creatinine kinase. Like GSD type 0 and type III, gluconeogenesis is preserved, and management is similar. Of note, this form of GSD is considered to exhibit a milder disease course compared to other types.

#### 2.5.5. Recent Updates in Management

More recently, medium chain triglyceride (MCT) oil has been explored in treatment of GSD type I. It has also been used in GSD type III and VI [37,38]. MCT oil has been found to inhibit de novo lipogenesis and slow glucose uptake [39]. When used as an adjunct to diet modification, MCT oil led to a significant reduction in hypoglycemia, triglycerides, and uric acid levels [39]. Gene therapy involving viral-mediated transfer of glucose-6-phosphatase is also currently being evaluated in maintaining euglycemia and reducing dependence on cornstarch, with promising results in a phase II trial [40].

### 2.6. Non-Islet Cell Tumor Hypoglycemia

Non-islet cell tumor hypoglycemia (NICTH) has an estimated incidence of 1/1,000,000, which is ~4x less than that of insulinomas [41,42]. The etiology has been ascribed to insulin-like growth factor-II (IGF-II), which shares structural homology with insulin but has a ~10-fold lesser glucose lowering effect [43]. The majority of IGF-II is bound to IGF binding protein 3 (IGFBP-3), a large, tertiary complex that typically does not interact with the insulin receptor. However, in NICTH, there is excess production of a modified IGF-II known as big IGF-II. There is a much larger pool of unbound big IGF-II as compared to unbound IGF-II and if bound, big IGF-II preferentially binds to IGFBP-2. These smaller, binary complexes and the higher amount of free big IGF-II contribute to greater bioavailability, leading to fasting hypoglycemia by suppressing hepatic gluconeogenesis and glycogenolysis, increasing skeletal muscle and adipose tissue glucose uptake, and decreasing lipolysis [42,44].

NICTH is typically caused by either mesenchymal (41%) or epithelial solid tumors (43%) [43]. Examples of mesenchymal tumors include mesotheliomas, leiomyosarcomas, gastrointestinal stromal tumors, and fibrosarcomas. Overall, hepatocellular carcinomas and stomach cancers are the most common causes of NICTH and comprise ~16% and 8% of cases, respectively. Therefore, in a patient with cancer and fasting hypoglycemia, NICTH should be suspected.

NICTH diagnosis is supported by demonstrating non-insulin mediated hypoglycemia, with appropriately low levels of proinsulin, c-peptide, and insulin, and inappropriately low ketone levels. Measurement of total IGF-II alone is not necessarily helpful, as total levels may be normal, while measurement of free or big IGF-II may not be commercially available [42]. The ratio of IGF-II/IGF-I is currently the most helpful diagnostic tool. This is because tumor-produced IGF-II provides negative feedback inhibition to GH, which in turn decreases hepatic IGF-I production. In a study published by Fukada and colleagues, an elevated IGF-II/IGF-I ratio > 20 was found in 94% of patients with NICTH, while only 42% of patients had elevated IGF-II levels [45].

Although there is no standard of care for NICTH management, treatment is primarily targeted at tumor bulk resection [42]. These tumors are often large (70% are >10 cm) and/or already disseminated where complete resection may not be possible [45]. In a recent systematic review, surgical resection and glucocorticoid use were significantly associated with patient recovery [46]. Glucocorticoids alleviate hypoglycemia by stimulating gluconeogenesis and suppressing production of big IGF-II [43]. Other treatments such as tumor embolization, radiation, diazoxide, and somatostatin receptor (SSTR) analogs have not produced consistent results [46].

More recently, medications that interfere with the insulin signaling pathway have been considered to address hypoglycemia caused by NICTH. Alpelisib and copanlisib are two FDA-approved medications for metastatic breast cancer and work by inhibiting phosphoinositide 3-kinase (PI3K) [47]. PI3K is an enzyme that activates protein kinase B (AKT), which regulates glucose metabolism, among other intracellular functions [48]. AKT activation leads to increased glucose transporter 4 (GLUT4) expression and translocation, ultimately leading to increased glucose uptake. Hyperglycemia is a notable side effect and occurs in up to 63% of patients [47]. Lastly, protease inhibitors used in the treatment of HIV competitively inhibit GLUT4, leading to decreased glucose uptake and transient insulin resistance, both of which may be beneficial to treat NICTH-induced hypoglycemia [47].

## 3. Insulin-Mediated Hypoglycemia

### 3.1. Post-Bariatric Hypoglycemia

Post-bariatric hypoglycemia (PBH) is a known complication after Roux-en-Y gastric bypass (RYGB) or sleeve gastrectomy that usually develops more than a year after bariatric surgery. Patients experience a hyperinsulinemic response to meals, especially meals high in carbohydrates, leading to postprandial hypoglycemia and neuroglycopenic symptoms [49]. Prevalence of PBH is difficult to accurately ascertain, as epidemiologic reports use different criteria to identify hypoglycemia, but hypoglycemic symptoms have been reported in approximately one third of patients after RYGB or sleeve gastrectomy [49].

PBH pathophysiology is not completely understood but appears to result from structural and functional changes. Altered gastrointestinal anatomy promotes rapid transit of nutrients into the small intestine, where there is a higher density of L-cells responsible for producing incretin hormones [50,51]. This leads to higher postprandial glucagon-like peptide-1 (GLP-1) levels, which then stimulate insulin secretion [49]. In addition, increased expression of intestinal glucose transporters post-surgery may facilitate rapid nutrient absorption [52]. These factors likely contribute to early postprandial hyperglycemia, which stimulates an exaggerated insulin response. Decreased insulin clearance, blunted glucagon responses, increased serotonin levels, altered bile acid metabolism, and changes in microbiome composition have also been implicated in the complex pathophysiology of PBH [49,53,54]. The hypothesis that nesidioblastosis is associated with PBH pathophysiology is a controversial subject, as this histopathologic diagnosis has been made in patients with PBH [55]. Whether nesidioblastosis is an incidental finding or represents pre/postsurgical pancreatic changes is currently not clearly elucidated.

PBH is diagnosed clinically by obtaining a detailed patient history. Patients report postprandial hypoglycemia with neuroglycopenic symptoms often triggered by simple carbohydrates. Patients may also document hyperglycemia preceding the hypoglycemic episode. CGMs may assist in detecting patterns of postprandial hyperglycemia followed by subsequent hypoglycemia, although they are not FDA approved for diagnosis. It is often not necessary to perform a mixed meal test to confirm hyperinsulinemic hypoglycemia, but it may be performed when the diagnosis is not clear. An oral glucose tolerance test is not recommended, as this could lead to hypoglycemic seizures and is not an accurate test for diagnosing hypoglycemia [49]. If a patient with bariatric surgery is reporting fasting hypoglycemia, this is not typical for PBH and a prolonged fast to evaluate for insulinoma should be performed.

Medical nutrition therapy is foundational to overall PBH management with the purpose of reducing nutrient-induced hyperglycemia, which stimulates robust insulin secretion. Patients are counseled to avoid liquid intake with food and to eat mixed meals high in protein and fiber and low in carbohydrates (<30 g per meal of complex carbs and avoiding simple sugars). Uncooked corn starch can be added to meals to try to slow nutrient absorption [56]. To prevent glycemic “yo-yo’ing” that occurs by correcting hypoglycemia with solely simple sugars, patients with PBH are instructed to consume 10–15 g of simple sugars to correct hypoglycemia, followed by intake of protein/fat (e.g., cheese stick, peanut butter) [49].

To date, there is no FDA-approved medication for use in treating PBH. Acarbose, an oral α-glucosidase inhibitor, slows carbohydrate digestion and absorption and has been found to be an effective and affordable first option, although gastrointestinal side effects may limit patient tolerance [57]. Glucagon-like peptide-1 receptor agonists (GLP-1RA) are often tried next in those who fail or do not tolerate acarbose. GLP-1RA use in PBH is counterintuitive but has been found to reduce glycemic variability and improve hypoglycemia frequency by saturating the GLP-1Rs exogenously, which may prevent endogenous fluctuations [58]. Diazoxide and SSTR analogs have also been used with varying success [59,60,61].

More recently, clinical trials are investigating the use of sodium-glucose cotransporter-1 inhibitors (SGLT-1i), SGLT1/2 inhibitors, and GLP-1R antagonists. For instance, mizagliflozin is an oral selective SGLT-1 inhibitor that has undergone phase II trials [62]. In treated participants, it led to a postprandial increase in glucose nadir, a reduction in peak glucose and insulin secretion, and a reduction in frequency of hypoglycemic events [63]. Canagliflozin inhibits SGLT1 at doses > 200 mg [64]. The HypoBar I trial is an ongoing randomized controlled trial that compares canagliflozin, acarbose, and placebo in treating PBH [65]. Avexitide, a GLP-1R antagonist, is currently undergoing a phase III trial, with a completed phase II trial successfully demonstrating a postprandial increase in glucose nadir, a reduction in insulin peak, and a reduction in rate of mild–severe hypoglycemic events [66,67].

Should lifestyle and medical therapy fail to improve PBH, surgical options may be considered in severe cases. In the case of RYGB, case reports have demonstrated that enteral nutrition via gastrostomy tube placement in the remnant pouch reduced hypoglycemic symptoms [68,69]. Silastic ring or gastric band placement to delay nutrient transit led to hypoglycemia resolution in 92% of patients in a small case series [70]. RYGB reversal has a success rate of 76% [71]. Partial pancreatectomy has fallen out of favor, as it has produced inconsistent results with frequent recurrence of hypoglycemia [49].

### 3.2. Insulin Autoimmune Syndrome

Insulin autoimmune syndrome (IAS) is the third leading cause of hypoglycemia in Japan and has been associated with the presence of Human Leukocyte Antigen (HLA) DRB1*0406, which is more prevalent in Asian populations [72]. However, other environmental triggers may play a role, including certain medications and viruses [73]. These environmental exposures may then trigger a type IV hypersensitivity reaction, leading to the production of pathogenic insulin autoantibodies (IAA) as described below. Antecedent viral infections with measles, mumps, rubella, cocksakie B, and varicella zoster viruses have been implicated by stimulating a robust immune response [73]. Medications such as methimazole and α-lipoic acid (an over-the-counter antioxidant) have also been reported to have a relatively high association with IAS. Of note, IAA that develop after exogenous insulin analog administration rarely cause IAS, because they tend to be characterized by a lower insulin binding capacity and higher affinity [73].

The pathogenesis of IAS involves the production of IAA with high insulin binding capacity but low affinity [73]. In response to a meal, insulin is produced but becomes inactive when bound by circulating IAA. This initially may lead to significant postprandial hyperglycemia due to low insulin bioavailability. However, these IAA-insulin complexes then spontaneously dissociate, leading to increased free circulating insulin, which then causes late hypoglycemia. This mismatch between insulin bioavailability and blood glucose levels is central to the pathogenesis of IAS-induced hypoglycemia [73].

Clinically, patients present with postprandial hypoglycemia, although fasting hypoglycemia has been reported. As such, hypoglycemia timing may not be a sensitive or specific feature of the disease. Laboratory features that suggest IAS are consistent with that of other endogenous insulin-mediated conditions (i.e., elevated insulin/c-peptide/proinsulin and suppressed BHB). However, a distinctive feature is the presence of significantly elevated levels of insulin above 1000 pmol/L, which is uncommonly seen in other types of endogenous insulin-mediated conditions. IAA assays are not routinely and commercially available, and some have proposed other methods of diagnosis, including polyethylene glycol precipitation and insulin to c-peptide molar ratio measurement [73].

Despite diagnostic challenges, IAS appears to have a self-remitting course. In almost 200 cases reported between its description in 1970 to 1992, ~80% of IAS cases spontaneously resolved after one–three months of recurrent hypoglycemic episodes [74]. Due to its rarity, there is no standard of care, although discontinuation of the offending agent has been found to decrease hypoglycemic episodes [74,75]. IAS management involves lifestyle/dietary modification to reduce the stimulus for insulin secretion. Uncooked corn starch and small frequent meals with low glycemic index have been found to decrease the frequency of hypoglycemic episodes [76,77]. Immunosuppression with glucocorticoids, azathioprine, and rituximab have all been found to be helpful in severe cases, although their use is mostly limited to case reports, and long-term outcomes are unknown [73]. Nevertheless, there appears to be a low recurrence rate after spontaneous resolution, as ~95% of the almost 200 cases reported until 1992 had sustained remission 1 year later [74].

Latest research exploring IAS treatment modalities demonstrate promising use of plasma cell-directed therapy for severe IAS refractory to lifestyle or immunosuppressive therapy. Askeland and colleagues describe two cases of successful treatment of IAS where patients remained in remission more than 3 years post therapy [78]. The treatment plan involved the use of bortezomib, lenalidomide, and dexamethasone for three–five cycles, followed by an autologous stem cell transplant in one case and daratumumab as maintenance therapy in the other case. This is helpful for clinicians to know in case of IAS refractory to other lifestyle or immunosuppressive therapies as described above.

### 3.3. Insulin Secretagogue and Exogenous Insulin

There are two main categories of insulin secretagogues: sulfonylureas and meglitinides. These two classes bind to different domains on sulfonylurea receptors expressed on pancreatic β-cells, promoting closure of K_ATP_ channels and subsequent insulin release [18]. Although primarily used in the treatment of diabetes mellitus, these medications and exogenous insulin have been implicated in non-diabetic use cases. For example, factitious use is a known cause of non-diabetic hypoglycemia and is more common in patients who have access to these medications or have a medical educational background [79]. Insulin has anabolic effects on skeletal muscle, which make it an appealing but potentially dangerous performance enhancing drug for recreational bodybuilders [80].

Diagnosis of insulin and insulin secretagogue-induced hypoglycemia requires biochemical evidence of insulin-mediated hypoglycemia with a few unique features [1]. In the setting of exogenous insulin use and normal pancreatic function, endogenous insulin secretion is suppressed, as evidenced by low c-peptide and pro-insulin. Ketone production is suppressed, and hepatic glycogen stores are preserved. In contrast, in insulin secretagogue-induced hypoglycemia, endogenous insulin production is ongoing, as evidenced by inappropriately non-suppressed or elevated c-peptide, pro-insulin, and insulin levels. Similarly, ketone production is suppressed, and hepatic glycogen stores are preserved. As this biochemical profile of insulin secretagogue-induced hypoglycemia is identical to that of insulinoma or noninsulinoma pancreatogenous hypoglycemic syndrome, serum or urine testing for oral hypoglycemic agents is important to make an accurate diagnosis.

Latest research in the management of factitious disorder is limited, with most data generated from case reports or case series [81]. Ultimately, long-term treatment of hypoglycemia due to insulin secretagogue and exogenous insulin primarily involves addressing the underlying problem by stopping the offending medication. However, because factitious hypoglycemia involves an underlying mental health disorder, a multidisciplinary approach with psychiatry and psychology is recommended [82].

### 3.4. Insulinoma

Insulinomas are insulin-producing tumors that arise from pancreatic β-cells and have an annual incidence of <4 cases per million [41]. Most of the time, they are localized to the pancreas, but ~10–15% are metastatic [41]. Prognosis of indolent (i.e., nonmetastatic) insulinomas is excellent and survival rates range between 94 and 100% [41]. However, survival rates of patients with aggressive (i.e., metastatic) insulinomas are lower, ranging between 24 and 67% [41]. The majority of insulinomas are sporadic, but ~5–10% are inherited as part of multiple endocrine neoplasia type 1 (MEN1) syndrome, and even more as part of the neurofibromatosis type 1 and tuberous sclerosis phenotype [83,84]. These tumors appear to arise from genetic and epigenetic mutations [41]. The most common is an activating mutation in the YY1 gene, which is involved as a transcription activator of the insulin gene [41]. The mammalian target of rapamycin (mTOR) pathway has also been implicated in insulinoma tumorigenesis. This complex pathway is involved in cellular proliferation and signaling. Increased expression and activation of this pathway was discovered in insulinoma tumors and appears to be involved in upregulating tumor growth and insulin secretion [85].

Classically, patients with an insulinoma present with fasting hypoglycemia. However, this is neither sensitive nor specific, as 20% may also experience postprandial hypoglycemia [41]. Patients may report weight gain, as insulin has anabolic properties and inhibits lipolysis and glycogenolysis [86]. Biochemical evidence of endogenous insulin-mediated hypoglycemia is crucial, and a supervised 72 h fast is usually recommended as a standard diagnostic step. Of note, it is helpful to know that 95% of patients with insulinoma meet end-of-fast criteria by 48 h, but the fast may be extended to 72 h as 7% of patients with proven insulinomas developed hypoglycemia after 48 h [87,88]. Once endogenous insulin-mediated hypoglycemia is uncovered, localization with endoscopic ultrasound is the preferred imaging modality, due to its high sensitivity (81%) and specificity (90%) [41]. Localization can be challenging, as size and tumor behavior are factors that need to be considered. Approximately 30% of insulinomas are <1 cm and, as noted above, a small percentage can be multifocal [41]. As indolent insulinomas tend to express GLP-1Rs, this may be used to the clinician’s advantage, and GLP-1R PET scans have reasonably high sensitivity and specificity (87% and 94%, respectively). In contrast, aggressive insulinomas preferentially express somatostatin receptor subtype 2 (SSTR2) instead of GLP-1R, and, in such cases, SSTR-SPECT scans may be utilized [41].

Treatment approach depends on several factors, including tumor size, behavior, location, and comorbidities. For surgical candidates with an indolent insulinoma, localized surgical resection is recommended. Whipple procedures are generally reserved for insulinomas that extend beyond the pancreatic head [41]. In cases where patients are not appropriate surgical candidates and have an indolent insulinoma, radiofrequency ablation (RFA) or stereotactic body radiotherapy are promising options. For patients with an aggressive insulinoma that has metastasized, surgical debulking is a treatment modality, as it is associated with improvement in hypoglycemia [89]. The liver and lymph nodes are common sites of metastasis, and liver metastases may be successfully treated with tumor resection, RFA, and chemoembolization [89].

Medical management should be considered in patients who are awaiting surgery, nonsurgical candidates, or in whom invasive procedures have not yielded significant benefit. Diazoxide, a β-cell K_ATP_ channel agonist, is FDA approved for insulinoma-induced hypoglycemia. Agonism of the K_ATP_ channel leads to its opening and subsequent cellular hyperpolarization, which then inhibits calcium influx and insulin release [90]. In a cohort study, approximately 60% of patients with an insulinoma reported resolution of hypoglycemic symptoms with seven years of diazoxide use [91]. Side effects such as fluid retention, hirsutism, and thrombocytopenia often limit its use. SSTR analogs have been used with a notable degree of success and may be considered as first line for metastatic insulinomas [41]. As insulinomas are known to express SSTR2, SSTR2 agonists such as octreotide and lanreotide have not only been shown to reduce hypoglycemia but also assist with tumor regression [92]. SSTR5 is the second most expressed receptor in insulinomas, and pasireotide, an analog with higher affinity for SSTR5, has been associated with a median progression-free survival of 11 months [93].

More recently, relatively newer medications have been explored as potential treatment modalities. Antitumoral treatment with peptide receptor radionuclide therapy (PRRT) was found to improve hypoglycemia and progression-free survival. Luthera was FDA approved in 2018 for SSTR-positive gastrointestinal neuroendocrine tumors and is a composed of radioactive 177-Lutetium paired with DOTATATE, a peptide SSTR agonist [92]. Concurrent use of this medication with octreotide led to significant improvement in progression-free survival and mortality risk reduction compared to octreotide alone [94]. Hypoglycemia reduction has also been noted as early as one–two cycles of treatment [95]. Reported side effects include gastrointestinal upset and bone marrow suppression. Other antitumoral options include everolimus and sunitinib, which may be considered in the setting of disease progression despite surgical and/or medical therapies discussed above. As noted earlier, the mTOR pathway is involved in insulinoma pathogenesis, and mTOR inhibitors such as everolimus have been explored as a treatment option with some success. Everolimus was found to downregulate insulin secretion by inhibition of the mTORC1/S6K pathway with subsequent improvement in hypoglycemia observed within 3–14 days of starting treatment [92]. Sunitinib is a multi-target receptor tyrosine kinase inhibitor and its use in metastatic insulinoma treatment is limited to case reports. In one published by Chen and colleagues, sunitinib stabilized tumor growth after two years of treatment but was noted to worsen hypoglycemia, an effect attributed to the drug’s ability to enhance glucose-dependent insulin secretion and reduce insulin clearance [96,97,98].

### 3.5. Noninsulinoma Pancreatogenous Hypoglycemic Syndrome

The incidence of noninsulinoma pancreatogenous hypoglycemic syndrome (NIPHS) is difficult to ascertain, but is considerably rarer than insulinomas with an estimated annual incidence of 0.5–5% [55]. It is important to mention that NIPHS is not synonymous or interchangeable with nesidioblastosis. Strictly speaking, nesidioblastosis is a histopathological term with specific major and minor criteria [99]. Major criteria include multiple β-cells with a macronucleus, abundant cytoplasm, and histopathologic and immunohistochemical exclusion of insulinoma. Minor criteria include irregular islet hypertrophy and hyperplasia [99]. Nesidioblastosis may be found in a wide variety of disorders and may occur without evidence of hyperinsulinemia or hypoglycemia [55]. In contrast, NIPHS remains a clinical diagnosis that depends on subjective history, biochemical evidence, dynamic testing, and functional imaging [55].

NIPHS pathophysiology is also not fully elucidated. However, central to available theories is the concept of augmented glucose-induced insulin secretion. It has been hypothesized that patients with NIPHS have a higher rate of basal insulin secretion and that the β-cells contain a higher amount of insulin stores. Other plausible mechanisms include activating mutations of glucokinase enzyme and higher (i.e., less negative) resting membrane potential threshold for glucose-induced K_ATP_ channel closure and cellular depolarization [55]. Lastly, pancreatic β-cells appear to have a higher density of GLP-1R in this condition, which may augment insulin sensitivity and secretion [100]. This is important because this may be exploited to aid in NIPHS diagnosis and treatment.

Patients with NIPHS usually present with postprandial hypoglycemia due to a hyperinsulinemic response to food, especially simple carbohydrates. NIPHS diagnosis should involve steps to exclude an insulinoma (72 h fast and imaging) as well as other causes of hypoglycemia. Seventy-two hour fasts are expected to be negative, although there has been reports of positive testing in such patients [55]. More recent research suggests that functional imaging with 68Ga-DOTA-Exendin-4 PET/CT is less invasive and may be just as sensitive as selective arterial calcium stimulation testing (SACST) in differentiating between insulinomas vs. NIPHS [100]. This imaging modality couples exendin-4, a GLP-1RA with radioactive tracer, allowing for demonstration of multifocal pancreatic involvement, a hallmark of NIPHS [100].

Treatment of hypoglycemia associated with NIPHS involves nutritional modification consisting of mixed meals high in protein and low in carbohydrates to assist with reduction in postprandial insulin secretion. Medical therapeutic options include acarbose, diazoxide, and SSTR analogs. In severe or refractory cases, NIPHS management may include either partial or subtotal pancreatectomy, which is helpful in up to 50% of cases [55,100]. More recently, other treatments have been explored in experimental contexts with animal models [55]. By coupling exendin-4 with a photosensitizer, Boss et al. demonstrated the selective destruction of lesions that express GLP-1R [101]. This is beneficial because it allows for the sparing of exocrine function, which may be affected after total/partial pancreatectomy.

## 4. Non-Diabetic Medications

Many non-diabetic medications have been associated with hypoglycemia. In a systematic review published by Murad and colleagues in 2009, they noted 109 medications with very-low-to-low quality of evidence, no medications with high quality of evidence, and seven medications with moderate quality of evidence [102]. Medications with moderate quality of evidence supporting their association with hypoglycemia are reviewed below, while medications with very-low-to-low quality of evidence are excluded (Table 4). The treatment of hypoglycemia primarily involves discontinuation of the offending drug. Acute treatment of hypoglycemia involves oral intake of glucose or intravenous dextrose if the patient is unable to safely swallow.

## 5. Conclusions

In conclusion, hypoglycemia is a clinical and biochemical diagnosis based on the fulfillment of Whipple’s triad. In evaluating hypoglycemia, a key step is to correlate symptoms with plasma glucose, as hypoglycemia mimics exist. The differential diagnosis of hypoglycemia remains broad, but a systematic, judicious approach enables the clinician to make an accurate diagnosis or at the very least, narrow the differential. The patient history, physical exam, and medication review are crucial aspects of the evaluation, as they provide important clues to certain causes of hypoglycemia. However, one should not make early diagnostic assumptions by solely depending on the timing of hypoglycemia, as it may not be a sensitive nor specific feature of the disease. Lastly, while the goal of hypoglycemia treatment is the restoration of euglycemia and eliminating associated symptoms, successful long-term management depends on identifying and addressing the underlying cause.

## Figures and Tables

**Table 1:** Symptoms associated with hypoglycemia.

Neurogenic	Neuroglycopenic
Diaphoresis Tingling Anxiety Tremulousness Palpitations Anxiety	Sensation of warmth Weakness Mental fogginess/confusion Fatigue Seizures Coma

**Table 2:** Insulin-mediated and non-insulin-mediated causes of hypoglycemia.

*Insulin-Mediated Hypoglycemia*	*Non-Insulin-Mediated Hypoglycemia*
**Exogenous** Exogenous insulin (including surreptitious use)	Critical illness
	Adrenal insufficiency
**Endogenous** Insulin secretagogues (sulfonylureas, meglitinides) Insulinoma Noninsulinoma pancreatogenous hypoglycemic syndrome Post-bariatric hypoglycemia Insulin autoimmune syndrome Non-diabetic medications	**Nutritional and metabolic disorders** Malnutrition and starvation Alcohol Glycogen storage diseases
	Non-islet cell tumors
	Non-diabetic medications Unripe ackee fruit

**Table 3:** Glycogen storage diseases associated with hypoglycemia.

	Etiology (Gene)	Pathogenesis	Clinical Features	Management
**GSD Type 0**	**Type 0a**: glycogen synthase 1 deficiency (GYS1) **Type 0b**: glycogen synthase 2 deficiency (GYS2)	Inability to convert glucose into glycogen. Gluconeogenesis preserved.	Fasting ketotic hypoglycemia Postprandial hyperglycemia **Type 0a**: Exercise intolerance Cardiomyopathy	Avoid fasting Frequent high protein meals Uncooked cornstarch
**GSD Type I**	**Type Ia**: Glucose-6-phosphatase α deficiency (G6PC) **Type 1b**: Glucose-6-phosphate transporter deficiency (SLC37A4)	Inability to catalyze final step of glycogenolysis and gluconeogenesis. Gluconeogenesis not preserved.	Doll-like facies Fasting ketotic hypoglycemia Hepatomegaly High triglycerides Lactic acidosis Hyperuricemia **Type 1b**: Neutropenia Recurrent infections Inflammatory bowel disease	Continuous enteral nutrition Uncooked cornstarch
**GSD Type III**	Glycogen debranching enzyme deficiency (AGL)	Inability to hydrolyze α-1,6-glycosidic bonds, causing abnormal glycogen accumulation. Gluconeogenesis preserved.	Fasting ketotic hypoglycemia Hepatomegaly High cholesterol High AST, ALT, CK *	Avoid fasting Frequent high protein meals Uncooked cornstarch
**GSD Type VI**	Hepatic glycogen phosphorylase deficiency (PYGL)	Inability to hydrolyze glycogen. Gluconeogenesis preserved.	Fasting ketotic hypoglycemia Hepatomegaly High cholesterol Normal CK	Treatment often not needed, as improves with age

* Aspartate aminotransferase (AST), alanine aminotransferase (ALT), creatine kinase (CK).

**Table 4:** Non-diabetic medications and ackee fruit associated with hypoglycemia.

	Medication Class	Hypoglycemic Mechanism	Notes
Indomethacin	NSAID *	Unclear	Seen in premature infants treated with IV indomethacin for PDA * [103]. Not usually seen in adults, and may even cause hyperglycemia [104]
Clinafloxacin and Gatifloxacin	Fluroquinolone	β-cell K_ATP_ channel inhibition [105].	Unavailable for systemic use in the United States [106,107]
Pentamidine	Anti-protozoal used for *Pneumocystis jiroveci* prophylaxis and treatment [108]	β-cell toxicity and death, leading to release of preformed insulin [109]	Hypoglycemia tends to occur within the first few days of administration followed by hyperglycemia in the subsequent 1–3 months [109]
Quinine	Anti-malarial	β-cell K_ATP_ channel inhibition Voltage-dependent K channels inhibition, prolonging β-cell membrane depolarization [110]	Seen when used to treat patients with severe malaria, who are already at higher risk for hypoglycemia due to severe illness and depletion of hepatic glycogen stores by the parasite [111]
Cibenzoline	Class Ia antiarrhythmic	β-cell K_ATP_ channel inhibition [112]	FDA approved but mostly used in Japan [112]
Glucagon	Anti-motility during endoscopy	Induces hyperglycemia, which stimulates insulin secretion [113]	May occur 90–120 min after administration [113]
Unripe ackee fruit	Tropical fruit native to West Africa	Hypoglycin A and B are metabolized into MCPA *, which inhibits fatty acid oxidation and impairs gluconeogenesis [114]	Jamaican vomiting sickness/toxic hypoglycemic syndrome, characterized by hypoglycemia, acidemia, liver injury, and severe vomiting. In severe cases, it has led to seizures, coma, or death [114]

* NSAID—nonsteroidal anti-inflammatory drug; PDA—patent ductus arteriosus; MCPA—methylene cyclopropyl acetic acid.

## Data Availability

No new data were created or analyzed in this study. Data sharing is not applicable to this article.

## Supplementary Materials

The following are available online at https://www.mdpi.com/article/10.3390/jcm14134393/s1, Figure S1: Detailed step-by-step diagnostic algorithm for non-diabetic hypoglycemia.

## Abbreviations

The following abbreviations are used in this manuscript:

BG	Blood glucose
CGM	Continuous glucose monitor
FDA	Food and Drug Administration
ISO	International Organization for Standardization
NAD+	Nicotinamide adenine dinucleotide (oxidized form)
NADH	Nicotinamide adenine dinucleotide (reduced form)
ATP	Adenosine triphosphate
CKD	Chronic kidney disease
AI	Adrenal insufficiency
GSD	Glycogen storage disease
DNA	Deoxyribonucleic acid
AST	Aspartate aminotransferase
ALT	Alanine aminotransferase
CK	Creatine kinase
G6P	Glucose-6-phosphate
GDE	Glycogen debranching enzyme
MCT	medium chain triglyceride
NICTH	Non-islet cell tumor hypoglycemia
IGF	Insulin-like growth factor
IGFBP	IGF binding protein
SSTR	Somatostatin receptor
PI3K	Phosphoinositide 3-kinase
AKT	Protein kinase B
GLUT4	Glucose transporter 4
HIV	Human Immunodeficiency Virus
PBH	Post-bariatric hypoglycemia
RYGB	Roux-en-Y gastric bypass
GLP-1	Glucagon-like peptide-1
GLP-1RA	Glucagon-like peptide-1 receptor agonist
SGLT-1i	Sodium-glucose cotransporter-1 inhibitor
IAS	Insulin autoimmune syndrome
HLA	Human Leukocyte Antigen
IAA	Insulin autoantibodies
MEN1	Multiple endocrine neoplasia type 1
mTOR	Mammalian target of rapamycin
PET	Positron emission tomography
SPECT	Single-photon emission computed tomography
RFA	Radiofrequency ablation
PRRT	Peptide receptor radionuclide therapy
NIPHS	Noninsulinoma pancreatogenous hypoglycemic syndrome
SACST	Selective arterial calcium stimulation testing
T1DM	Type 1 diabetes mellitus
NSAID	Nonsteroidal anti-inflammatory drug
PDA	Patent ductus arteriosus
MCPA	Methylene cyclopropyl acetic acid
