# Supplementary figures and images for: Non-Diabetic Hypoglycemia: Evaluation and Management in Adults
Source: J Clin Med. 2025 Jun 20;14(13):4393. doi: 10.3390/jcm14134393 (PMC12250112; doi:10.3390/jcm14134393)

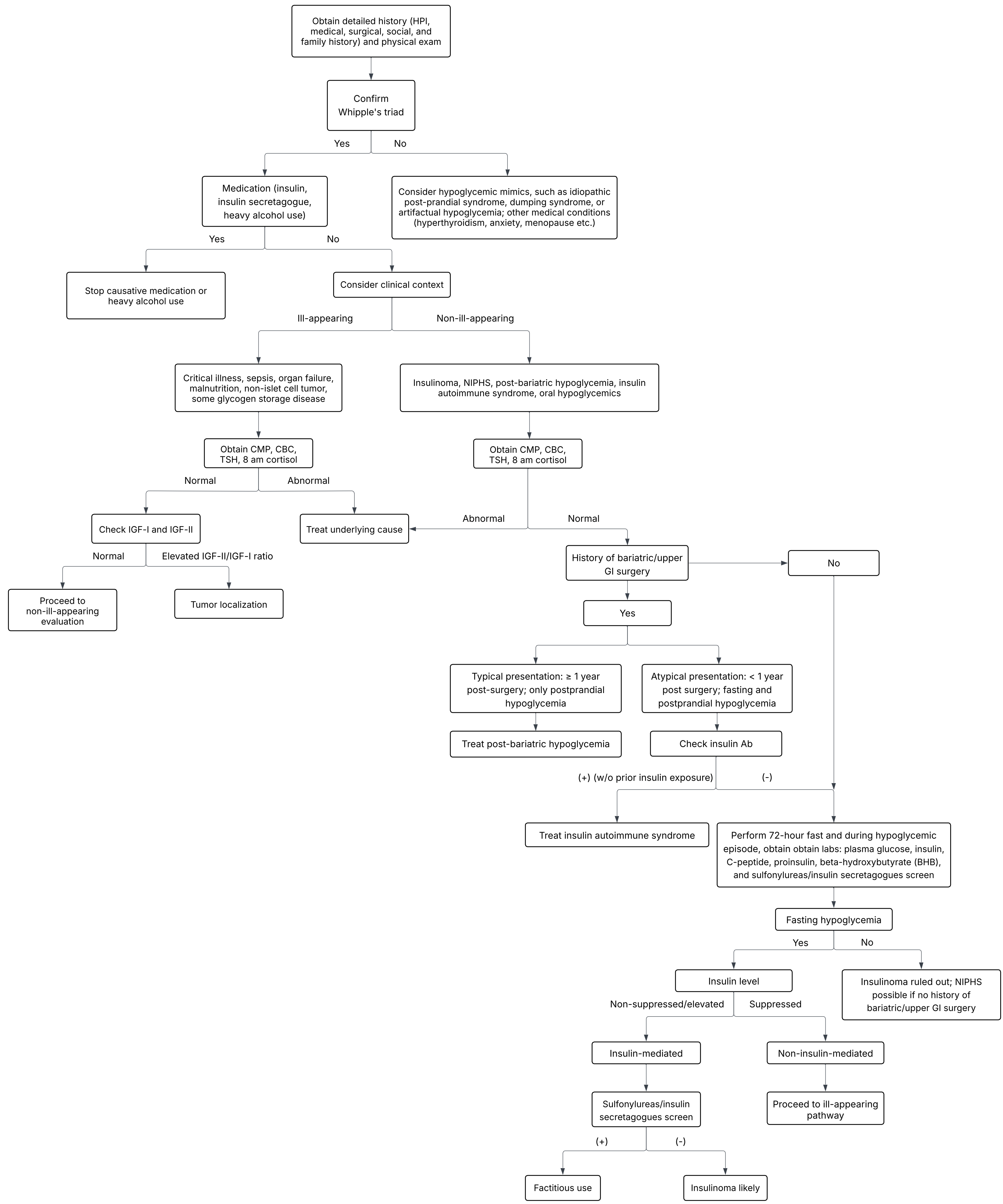

Supplement: Supplementary file 1 [file jcm-14-04393-s001.zip › jcm-3616011-supplementary.pdf]
